# Development of a Replication-Deficient Bacteriophage Reporter Lacking an Essential Baseplate Wedge Subunit

**DOI:** 10.3390/v16010008

**Published:** 2023-12-20

**Authors:** Jose Gil, John Paulson, Henriett Zahn, Matthew Brown, Minh M. Nguyen, Stephen Erickson

**Affiliations:** 1 Laboratory Corporation of America Holdings, Los Angeles, CA 90062, USA; gilj@labcorp.com; 2 Laboratory Corporation of America Holdings, New Brighton, MN 55112, USA; paulsoj@labcorp.com (J.P.); zahnh@labcorp.com (H.Z.); nguyem5@labcorp.com (M.M.N.); 3 Laboratory Corporation of America Holdings, Burlington, NC 27215, USA; browm49@labcorp.com

**Keywords:** bacteriophage, phage-based detection, *Salmonella enterica*, luciferase reporter phage, replication-deficient phage, baseplate wedge subunit, synthetic phage

## Abstract

Engineered bacteriophages (phages) can be effective diagnostic reporters for detecting a variety of bacterial pathogens. Although a promising biotechnology, the large-scale use of these reporters may result in the unintentional release of genetically modified viruses. In order to limit the potential environmental impact, the ability of these phages to propagate outside the laboratory was targeted. The phage SEA1 has been previously engineered to facilitate food safety as an accurate and sensitive reporter for *Salmonella* contamination. In this study, homologous recombination was used to replace the expression of an essential baseplate wedge subunit (gp141) in SEA1 with a luciferase, NanoLuc^®^. This reporter, referred to as SEA1Δgp141.NL, demonstrated a loss of plaque formation and a failure to increase in titer following infection of *Salmonella*. SEA1Δgp141.NL was thus incapable of producing infectious progeny in the absence of gp141. In contrast, production of high titer stocks was possible when gp141 was artificially supplied in trans during infection. As a reporter, SEA1Δgp141.NL facilitated rapid, sensitive, and robust detection of *Salmonella* despite an inability to replicate. These results suggest that replication-deficient reporter phages are an effective method to obtain improved containment without sacrificing significant performance or the ease of production associated with many phage-based diagnostic methods.

## 1. Introduction

The genetic modification of bacteriophages (phages) has been used to support the development and advancement of phage-based biotechnologies. Synthetic phages have been engineered with improved capacity to disperse bacterial biofilms, digest bacterial DNA, and neutralize bacterial defense mechanisms [1,2,3]. In addition to targeted enhancements, genetic manipulation has also been used to address the intrinsic limitations of otherwise promising phage candidates. For example, the first synthetic phages used in human therapy were temperate mycobacterial phages modified to achieve a strictly lytic life cycle [4]. Furthermore, phages with suboptimal host ranges have been improved through the engineering of receptor-binding proteins, eliminating cross-reactivity and improving coverage of the target bacterial pathogen [5,6]. Due to their versatility, synthetic phages represent an attractive solution to the cumbersome search for natural phages with ideal properties.

In addition to improving therapeutic applications, genetic engineering has also led to the emergence of promising phage-based diagnostics. Phages have now been modified to encode a variety of reporter genes, including luciferases, fluorescent proteins, alkaline phosphatase, and β-galactosidase [7,8,9,10]. When encoded in the phage genome, the production of these reporters occurs alongside phage proteins during infection, facilitating the specific detection of viable bacterial hosts. One particularly promising reporter phage design utilizes the engineered commercially available luciferase NanoLuc^®^. NanoLuc^®^ is an attractive candidate as a reporter gene due to its smaller size and increased brightness when compared to historically available luciferases [11]. In recent years, NanoLuc^®^-encoding phage reporters have been developed to detect a variety of bacterial pathogens, including those relevant to food safety, such as *Salmonella enterica* and *Listeria monocytogenes*, and those with widespread clinical significance, such as *Staphylococcus aureus* and *Mycobacterium tuberculosis* [12,13,14,15]. Thus, when properly designed, genetically modified phages have facilitated the development of rapid, sensitive, and robust bacterial diagnostics.

Although synthetic phages have significant potential, widespread commercial use could result in the accidental release of genetically modified viruses into the environment. These concerns have been raised previously, although the definitive impact of a release cannot be easily predicted, depending heavily on the nature of the engineering [16,17,18]. However, it is clear that the capacity of synthetic phages to replicate, persist, and potentially continue to produce their genetic payload outside of the intended use contributes to the magnitude of these concerns. To this end, synthetic replication-deficient or “biocontained” phages have previously been suggested as a potential generalizable solution for phage-based biotechnologies [16,17,18]. With this approach, an essential structural gene is removed from the phage genome, resulting in a phage incapable of producing infectious progeny. The production of these phages is achieved in the laboratory by artificially supplying the deleted gene in trans. This complementation strategy has shown success using essential capsid and tail proteins [17]. Critically, replication-deficient phages produced by this method were even found to possess comparable therapeutic potential [17]. The viability of this approach has yet to be explored in diagnostic applications of phage reporters.

*Salmonella* is a major foodborne pathogen, annually linked to nearly 100-million infections worldwide and over 100,000 deaths [19]. In the United States of America alone, salmonellosis results in billions of dollars of detrimental economic impact [20]. Due to this significance, the accurate and sensitive detection of *Salmonella* in foods is essential to ensure food safety and public health. To address this concern, a phage-based diagnostic method, known as the PhageDx™ *Salmonella* assay, was developed to facilitate *Salmonella* detection in food matrices [12,21]. Over the course of several studies, the PhageDx™ *Salmonella* assay has demonstrated excellent performance in both broth culture and across multiple food matrices, including ground turkey, powdered infant formula, hydroponic curly lettuce, and Brazilian poultry products [12,21,22,23]. The detection of contamination by this method is mediated by NanoLuc^®^-encoding *Salmonella* reporter phages, engineered from two natural phages isolated from wastewater [12,24]. One of these phage reporters, SEA1.NL, has superb inclusivity and provides broad coverage of *S. enterica*, yielding a positive signal over the background from 99% of tested strains [12]. The importance of phage replication in the context of SEA1.NL’s performance was unknown.

The purpose of this study was to assess the efficacy of replication-deficient phages within the context of phage diagnostics, specifically NanoLuc^®^-encoding reporters. To this end, recombination was used to replace gp141 expression in SEA1 with NanoLuc^®^ expression, creating SEA1Δgp141.NL. SEA1’s gp141 is the homolog of an essential baseplate wedge subunit in the well-studied *Escherichia coli* phage T4, gp53 [25,26]. In each T4 virion, six copies of gp53 join adjacent wedge complexes and are required to stabilize the hexagonal foundation of the phage baseplate [27,28,29]. As expected, the loss of gp141 rendered SEA1 incapable of producing infectious progeny following the infection of its native bacterial host. Importantly, the supplementation of gp141 in trans could complement this defect and facilitated laboratory production of high titer stocks suitable for downstream diagnostic testing. Despite an inability to replicate, SEA1Δgp141.NL remained fully capable of detecting *S. enterica* contamination, producing a significant burden-dependent luminescent signal over background. Overall, this study highlights the potential of the replication-deficient phage for the development of bacterial diagnostics with improved containment and real-world safety profiles.

## 2. Materials and Methods

### 2.1. Bacterial Strains and Bacteriophage Stocks

Bacterial stocks were obtained from the American Type Culture Collection (ATCC) (Manassas, VA, USA). Specifically, ATCC 27869, a strain of *Salmonella enterica* subsp. *enterica* var. Newport, was used throughout this study as a representative *S. enterica* host. *Salmonella* was routinely cultured overnight in tryptone soy broth (TSB) (Oxoid, Hampshire, UK) at 37 °C with shaking at 225 revolutions per minute unless otherwise indicated. As needed, tryptone soy agar (TSA) plates were prepared with a final concentration of 1.5% agar. When appropriate for plasmid maintenance, transformed bacterial strains were grown in media supplemented with either ampicillin (100 µg/mL) or carbenicillin (100 µg/mL).

The development, purification, and production of SEA1.NL has been reported elsewhere in detail [12]. SEA1∆gp141.NL was generated in the current study and the genetic engineering of this recombinant is described below in Section 2.3. and Section 2.4. Production of purified high titer stocks of SEA1∆gp141.NL, typically greater than 1 × 10^11^ pfu/mL, was achieved through a density gradient as previously described for SEA1.NL, with one exception. In place of the wild-type strain, *S. enterica* transformed with the gp141 complementation plasmid (pUC57.Comp.gp141), described below in Section 2.3, was used as the host strain for SEA1∆gp141.NL to permit phage replication.

### 2.2. Genetic Comparison of SEA1 and T4

The annotated genome of the *Salmonella* phage SEA1 is now available on GenBank^®^ (OQ927978). This sequence was analyzed alongside the genome of the well-studied *Escherichia coli* phage T4, which is also available on GenBank^®^ (NC_000866). Sequence comparisons were initially performed using Basic Local Alignment Search Tool (BLAST) [30]. Genomes were compared using Nucleotide BLAST, while homologs of T4’s gp53 were obtained using Protein BLAST. To determine protein similarity, a Needleman–Wunsch alignment of the amino acid sequence for each T4, and the SEA1 homolog was performed using the European Molecular Biology Open Software Suite (EMBOSS) Needle program (www.ebi.ac.uk access on 2 November 2023) [31,32].

### 2.3. Design of Homologous Recombination and Complementation Plasmid

In order to exchange the gene encoding gp141 with the gene encoding NanoLuc^®^ in SEA1, a synthetic plasmid with a homologous recombination cassette engineered to facilitate this recombination was required. To target recombination, two 500 bp flanks of homology to the DNA sequences immediately upstream and downstream of the gene encoding gp141 were used. Between these flanks, a bacterial promoter containing the −10 consensus sequence (TATAAT) of the σ^70^ promoter and a Shine–Dalgarno sequence (AGGAGGT) preceded a *Salmonella* codon-optimized gene encoding NanoLuc^®^. These promoter and codon-optimized gene sequences are identical to those used previously for the generation of SEA1.NL [12]. This cassette was commercially synthesized and inserted into the pUC57 plasmid backbone by GeneWiz (South Plainfield, NJ, USA), creating pUC57∆gp141.NL. This plasmid was transformed into *S. enterica* by electroporation using protocols suitable for *Salmonella* [12].

Since gp141 was expected to be essential for replication, a plasmid was designed to supply this protein in trans and complement any intrinsic growth defect of the gp141-deficient phage. To this end, a bacterial promoter containing the −35 (TTGACA) and −10 (TATAAT) consensus sequences of the σ^70^ promoter and a Shine–Dalgarno site (AGGAGGT) was placed upstream of the gene encoding gp141 from SEA1. Sequences were commercially synthesized and inserted into the pUC57 plasmid backbone by GeneWiz (South Plainfield, NJ, USA), creating pUC57.Comp.gp141. This plasmid was transformed into *S. enterica* by electroporation as done previously.

### 2.4. Genetic Engineering of SEA1∆gp141.NL

Genetic engineering of SEA1∆gp141.NL was performed through homologous recombination. First, *S. enterica* transformed with pUC57∆gp141.NL, described above in Section 2.3, was grown to a bacterial concentration of 1 × 10^7^ cells/mL, as measured by a hemocytometer. A sample (500 µL) was removed and infected with SEA1 at a multiplicity of infection of 0.1 for three hours at 37 °C. Following infection and recombination, SEA1∆gp141.NL was isolated from this lysate on the basis of NanoLuc^®^ production by serially performing limiting dilution enrichments, similar to the isolation of prior luciferase phage reporters [12,14,33]. For this recombinant, enrichments were performed by infecting the complementation strain of *S. enterica* transformed with pUC57.Comp.gp141, described above in Section 2.3., for two to four hours at 37 °C. A total of five rounds of enrichment were performed prior to plaque isolation.

After serial enrichment, individual plaques were obtained using a standard double-layer agar technique and screened for NanoLuc^®^ production as follows. Plaques were first picked into 500 µL of TMS (50 mM Tris–HCl pH 7.8, 10 mM MgCl_2_, and 300 mM NaCl) and 20 µL of chloroform. This mixture was pelleted by centrifugation for 2 min at 6800× *g* and 450 µL of the supernatant spin filtered (Ultrafree^®^–MC centrifugal 0.45 µM filter, MilliporeSigma, Burlington, MA, USA). A 10 µL portion of this filtrate was transferred to wells of a 96-well strip plate containing 200 µL of a hundred-fold diluted overnight culture of the gp141-complementing *S. enterica* host strain. After a 3 h incubation at 37 °C, the luciferase activity of each well was assessed by adding 50 µL of a detection master mix. For each well, this detection master mix was prepared with 50 µL of Nano–Glo^®^ buffer (Promega Corporation, Madison, WI, USA), and 1 µL of Nano–Glo^®^ substrate (Promega Corporation, Madison, WI, USA). Luminescence was quantified on a GloMax^®^ 96 Microplate Luminometer (Promega Corporation, Madison, WI, USA) with a 3 s integration time. After their identification, NanoLuc^®^-positive plaques were diluted and plated once again to obtain a second round of single plaques. Plaque isolation and passaging of NanoLuc^®^-positive suspensions was repeated a total of five times to ensure the purity and isolation of SEA1∆gp141.NL.

### 2.5. Phage DNA Preparation and PCR Confirmation

To obtain phage DNA suitable for PCR analysis, stocks were first diluted in TMS to approximately 6 × 10^10^ PFU in a 750 µL volume. After heating (90 °C) for 2 min, 20% sodium dodecyl sulfate, 0.1 M ethylenediaminetetraacetic acid, and 20 mg/mL proteinase K were added to achieve final concentrations of 0.1%, 5 mM, and 53 ng/µL, respectively. This mixture was incubated for 1 h at 50 °C, and the released DNA was purified by three rounds of phenol chloroform extraction. DNA was precipitated with alcohol, washed, air-dried, and resuspended in molecular grade water for PCR analysis.

PCR was performed using the Invitrogen™ Platinum™ *Taq* DNA Polymerase (Thermo Fisher Scientific, Waltham, MA, USA), largely as per manufacturer instructions. Approximately 100 ng of DNA were used for each reaction. Detection of the gene encoding gp141 was performed using the forward primer (5′-AGGATTTGTATCGCACCATCTC-3′) and the reverse primer (5′-AAGCAATCTCCATCTCTCTGATT-3′). Based upon these sequences, an annealing temperature of 50 °C was selected. After 30 cycles, 5 µL of PCR product were diluted with 10 µL of molecular grade water and combined with 3 µL of 6X loading dye (New England Biolabs, Ipswich, MA, USA). Diluted PCR product (10 µL) was added to a 1.5% agarose gel and run at 100 V for approximately 90 min. DNA bands were visualized with SYBR Safe DNA Gel Stain (Thermo Fisher Scientific, Waltham, MA, USA). The O’GeneRuler 1 kb Plus DNA ladder (Thermo Fisher Scientific, Waltham, MA, USA) was also included for band size determination as per manufacturer instructions. Images were captured using a Gel Doc™ EZ Imager (Bio–Rad Laboratories, Hercules, CA, USA) running Image Lab 5.2.1 Software (Bio–Rad Laboratories, Hercules, CA, USA).

### 2.6. Evaluation of Spot Lysis and Spot Plaque Formation

A double-layer agar method, described previously, was used to evaluate phage plaque formation and spot lysis [6]. Briefly, bacterial cultures of *S. enterica* and *S. enterica* transformed with the gp141 complementation plasmid (pUC57.Comp.gp141) were grown to exponential phase. This culture (100 µL) was combined with 3 mL of molten semi-solid (0.5% agar) TSA, mixed, and poured atop TSA plates. Plates were allowed to solidify at room temperature before use. Phage stocks were prepared at 1 × 10^11^ pfu/mL and then serially 10-fold diluted eight times. For each indicated phage, nine samples consisting of the initial stock and eight dilutions were spotted (4 µL) onto the prepared double-layer agar plate. Spotted phages were allowed to absorb briefly at room temperature and incubated overnight at 37 °C. The next day, plates were removed and photographed using a Gel Doc™ EZ Imager (Bio–Rad Laboratories, Hercules, CA, USA) running Image Lab 5.2.1 Software (Bio-Rad Laboratories, Hercules, CA, USA).

### 2.7. One-Step Growth Curve

To evaluate phage growth characteristics, one-step growth curves were performed [34]. Bacterial cultures of *S. enterica* and *S. enterica* transformed with the gp141 complementation plasmid (pUC57.Comp.gp141) were diluted to approximately 2 × 10^8^ CFU/mL. Phage stocks of SEA1.NL and SEA1∆gp141.NL were diluted to 4 × 10^6^ PFU/mL. Infection was initiated by combining 900 µL of diluted bacterial culture with 100 µL of diluted phage preparation. After a 10 min adsorption period at 37 °C, 100 µL of this mixture were diluted into 9.9 mL of fresh warmed TSB and vortexed. This dilution was repeated in the same manner by once again transferring 100 µL into 9.9 mL of fresh warmed TSB. The infection was then allowed to proceed at 37 °C and monitored. At each indicated time point, beginning immediately after the 10 min adsorption and dilution series, a 100 µL sample of the twice-diluted phage infection was combined with 100 µL of indicated bacterial culture and 3 mL of molten semi-solid (0.3% agar) TSA. Once mixed, this solution was poured atop TSA plates, allowed to solidify, and incubated overnight at 37 °C before counting plaques. The limit of detection for this method is 100 total PFU in the final 10 mL dilution. The latent period was determined as the period up to an observable increase in phage titer. The burst size was calculated as the fold difference between the PFU during the latent period and the PFU after the growth curve reached a plateau.

### 2.8. NanoLuc^®^ Reporter Phage Assay

NanoLuc^®^ production was evaluated in a similar manner to prior studies [6,12,14]. *S. enterica* was grown to exponential phase and serially diluted to obtain concentrations between 100 CFU/mL and 1 × 10^8^ CFU/mL. Two sets of triplicate wells were prepared by adding 100 µL of either TSB (0 CFU) or each bacterial dilution to achieve the indicated burden per well in a 96-well plate. This was repeated for four separate 96-well plates to allow analysis at four distinct time points (30-, 60-, 90-, and 120-min post infection). Next, 10 µL of a working stock (1 × 10^7^ pfu/mL) of either SEA.NL or SEA1∆gp141.NL was added to each well. 96-well plates were sealed with cover film and incubated at 37 °C. At the indicated time points, a 96-well plate was removed and analyzed for luciferase activity by adding 65 µL of a detection master mix. For each well, this detection master mix was prepared with 50 µL of Nano–Glo^®^ buffer (Promega Corporation, Madison, WI, USA), 15 µL of Renilla lysis buffer (Promega Corporation, Madison, WI, USA), and 1 µL of Nano–Glo^®^ substrate (Promega Corporation, Madison, WI, USA). Luminescence was detected as relative light units (RLU) on a GloMax^®^ Navigator (Promega Corporation, Madison, WI, USA) with a 1 s integration time after a 3 min delay. Two back-to-back reads were performed (technical replicates) and averaged together to obtain the value for each well. For visualization and analysis, RLU values were log transformed, and the mean and standard deviation were graphed. At each time point, a two-way analysis of variance (ANOVA) with Dunnett’s multiple comparisons test was used to determine statistical significance (*p* < 0.05). For each phage reporter, the RLU values produced at the indicated bacterial burden were compared to background (0 CFU). An asterisk was used to indicate when a sample was significantly different from background. Statistical analysis was conducted with GraphPad Prism 10 (GraphPad Software, Boston, MA, USA).

## 3. Results

### 3.1. SEA1 Encodes a Homolog to gp53, T4’s Essential Baseplate Wedge Subunit

A proven strategy for generating stable replication-deficient viruses is through the deletion of an essential gene from the genome, thereby restricting viral replication to genetically modified hosts expressing the required gene in trans [35,36,37]. The diagnostic effectiveness of a replication-deficient reporter phage was unknown. To explore the importance of replication in this system, a replication-deficient NanoLuc^®^-encoding reporter of the broad host range *Salmonella* phage SEA1 was pursued. This phage, originally isolated from wastewater, can already be used as a sensitive reporter for *Salmonella* contamination following genetic engineering [12,24]. The complete genome sequence and annotation of SEA1 has recently been made available through GenBank^®^ (OQ927978).

**Figure 1:**
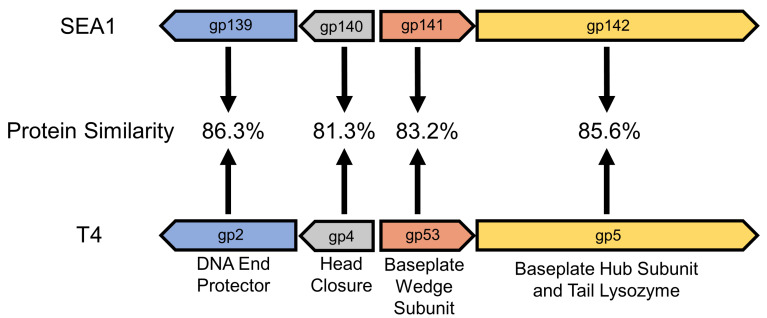
Homologs of *Escherichia* phage T4’s essential baseplate wedge subunit and nearby proteins are found in *Salmonella* phage SEA1. Colors distinguish each set of homologs between T4 (NC_000866) and SEA1 (OQ927978), with arrows indicating calculated protein similarity. Protein similarity was determined using EMBOSS Needle [32]. The protein product information from T4’s GenBank^®^ entry was included below each gene.

In order to identify a candidate essential gene, the sequence of SEA1 was investigated. SEA1 was found to have modest genomic similarity to the well-studied *Escherichia coli* phage T4, approximately 69% identity over 57% query coverage, by BLAST comparison [30]. Only roughly 20% of genes in T4 are described as essential [25,26]. Among these essential genes, the gene encoding gp53 in T4 proved to be an attractive candidate that had not been previously investigated for this purpose. Annotated as a baseplate wedge subunit, gp53 is structurally critical, mediating the formation of the phage baseplate by joining wedge complexes [27,28,29]. Thus, the absence of this single gene would be predicted to irreversibly prevent virion assembly and generate a stable replication-deficient phage. In addition, only six copies of gp53 are required per phage, indicating that modest expression in trans may be sufficient to achieve high titer production in gp53-expressing laboratory hosts [27,28,29]. Importantly, this locus appears relatively well-conserved at the protein level, and T4’s gp53 has 83.2% similarity to SEA1’s gp141, suggesting that gp141 would be a suitable candidate in SEA1 (Figure 1). Lastly, gp141 is a small protein (191 amino acids), similar to NanoLuc^®^ (171 amino acids), and thus exchanging the genes encoding these proteins would have a negligible effect on overall genome size [11].

### 3.2. Genetic Engineering of SEA1∆gp141.NL

**Figure 2:**
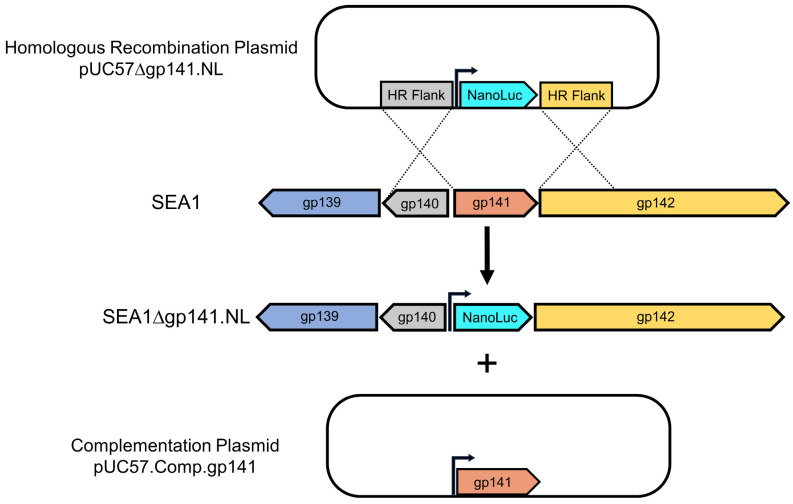
Homologous recombination was used to generate SEA1∆gp141.NL. Infection of *S. enterica* carrying the pUC57∆gp141.NL plasmid with SEA1 was performed at a multiplicity of infection of 0.1 to generate the desired recombinant, SEA1∆gp141.NL. After the initial infection, *S. enterica* carrying the complementation plasmid pUC57.Comp.gp141 was used to supply gp141 in trans to permit isolation and production of SEA1∆gp141.NL. Consistent with Figure 1, genes were assigned a color to aid in visualization. Arrows in the genomic sequence indicate sites where bacterial promoters were added.

To delete gp141 from SEA1 and simultaneously generate a NanoLuc^®^-encoding reporter, a genetic engineering strategy was devised (Figure 2). Following SEA1 infection of transformed *S. enterica* carrying pUC57∆gp141.NL, homologous recombination was expected to generate the desired recombinant, SEA1∆gp141.NL. Due to the anticipated replication-deficiency of this recombinant, purification and production were performed on a *S. enterica* host carrying a complementation plasmid, pUC57.Comp.gp141, to supply gp141 in trans. Barring this exception, the approach for isolating and manufacturing a NanoLuc^®^-positive phage was similar to prior studies [12,14]. High titer stocks of at least 1 × 10^11^ pfu/mL were obtained in this manner.

### 3.3. SEA1∆gp141.NL Is Deficient in Plaque Formation and Replication

**Figure 3:**
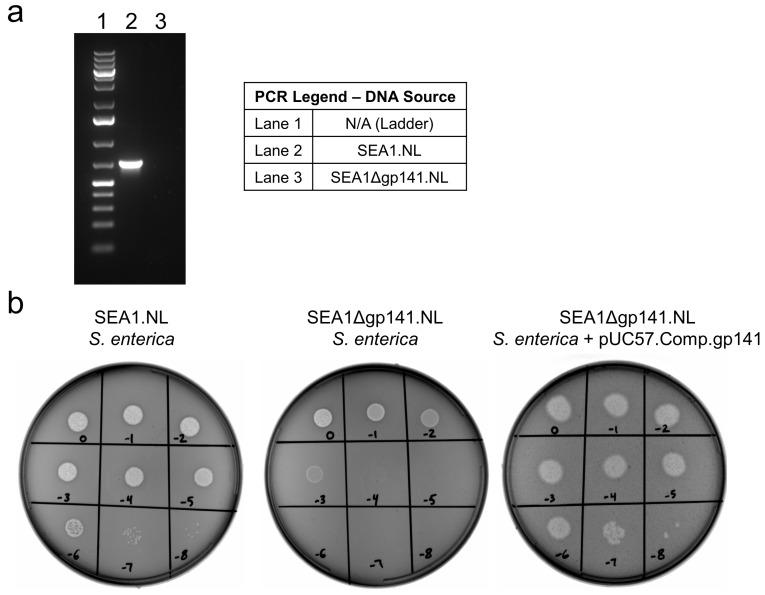
SEA1∆gp141.NL was confirmed by PCR and was deficient for plaque formation in the absence of gp141. PCR analysis (**a**) was performed on DNA preparations from SEA1.NL and SEA1∆gp141.NL using primers specific for gp141. Ladder refers to O’GeneRuler 1 kb Plus DNA ladder. An original uncropped gel image is provided elsewhere (Supplementary Figure S1). Plaque formation (**b**) and spotting activity was determined by spotting 4 µL of each serially diluted phage preparation onto a double-layer agar containing *S. enterica*. The number “0” refers to a working stock concentration of 1 × 10^11^ pfu/mL. Ten-fold serial dilutions of this stock are subsequently notated as 10^x^. When indicated, the *S. enterica* host carried the gp141 complementation plasmid pUC57.Comp.gp141.

Following isolation and propagation, SEA1∆gp141.NL was first confirmed to lack gp141 by PCR. Unlike the control SEA1.NL, purified DNA preparations from SEA1∆gp141.NL failed to generate a band with primers specific for gp141 (Figure 3a). An uncropped original gel image is available (Supplementary Figure S1). Due to the predicted importance of gp141, SEA1∆gp141.NL was expected to be replication-deficient on *Salmonella* hosts lacking the complementation plasmid. To confirm this expectation, phage stocks were serially diluted and spotted onto double-layer agar plates containing an overlay of semi-solid agar mixed with *S. enterica* (Figure 3b). As expected, a serial dilution of SEA1.NL yielded single plaques on *S. enterica*, a clear indicator of productive infection. In contrast, SEA1∆gp141.NL was deficient for plaque formation on this host. Importantly, plaque formation of SEA1∆gp141.NL was restored when conducted on a host strain expressing gp141 in trans. Unlike plaque formation, spot formation was observed with both SEA1.NL and SEA1∆gp141.NL at higher titers. This has been observed previously with replication-deficient phages, and spot formation is not dependent on productive infection [17]. Overall, these results demonstrate that SEA1∆gp141.NL is not capable of plaque formation without the assistance of a complementation host, indicative of, at a minimum, a substantial decrease in phage replication in the absence of gp141.

**Figure 4:**
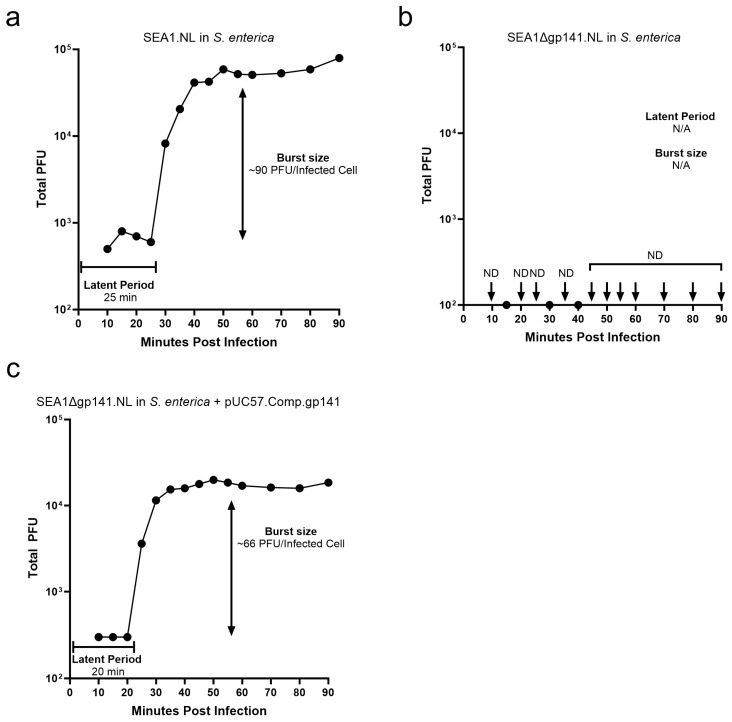
A one-step growth curve establishes gp141 as essential for SEA1 replication. Infection of a *S. enterica* with either SEA1.NL (**a**) or SEA1∆gp141.NL (**b**) was performed, and plaque-forming units (PFU) were monitored over a 90-min period. When indicated (**c**), *S. enterica* expressing gp141 in trans was similarly infected and monitored. To circumvent the deficiency in plaque formation, samples of SEA1∆gp141.NL (**b**,**c**) were plated on the gp141-expressing host strain to determine PFU. The latent period was defined as the time prior to an observed increase in phage titer. The burst size was defined as the fold difference between the average PFU during the latent period and average PFU following the plateau of growth. The *y*-axis was set at the limit of detection for the experiment (100 PFU). Samples that yielded no plaques and are thus below the limit of detection are marked with an arrow and indicated as not detected (ND). Individual plaque counts are provided elsewhere (Supplementary Table S1).

Although the absence of plaque formation was suggestive, this alone does not confirm the complete absence of replication. For example, the disruption of genes involved in host DNA degradation in T4 can result in significantly reduced burst sizes with only a handful of progeny produced per infected cell [38,39]. Importantly, a burst size of at least 10 has been suggested to be a requirement for reliable plaque formation [25]. In order to rule out the possibility of low levels of replication in the absence of gp141, a one-step growth curve was performed [34]. After infection of the *S. enterica* strain used in this study, SEA1.NL demonstrated a latent period of 25 min and a burst size of approximately 90 plaque forming units (PFU) per infected cell (Figure 4a). Similar values have been previously reported for T4 [40]. In contrast, SEA1∆gp141.NL failed to demonstrate an identifiable burst or latent period in this strain (Figure 4b). Importantly, although the infection and growth curve were performed using a standard *S. enterica* host, PFU were subsequently assessed on a strain expressing gp141 in trans. This is required to distinguish a lack of phage replication from simply a continued failure to form plaques. Despite this, only a single plaque was detected at three time points (15, 30, and 40 min), and no plaques were identified after 40 min. As seen previously, plaques were not detected when SEA1∆gp141.NL was plated on native *S. enterica* (Supplementary Table S1). Importantly, a one-step growth curve using SEA1∆gp141.NL on *S. enterica* expressing gp141 in trans demonstrated a restoration of replication with a latent period of 20 min and a burst size of approximately 66 plaque-forming units per infected cell (Figure 4c). These results support the replication-deficiency of SEA1∆gp141.NL and confirm that this phenotype is solely due to the absence of gp141.

### 3.4. Despite Replication-Deficiency, SEA1∆gp141.NL Remains an Effective Salmonella Reporter

**Figure 5:**
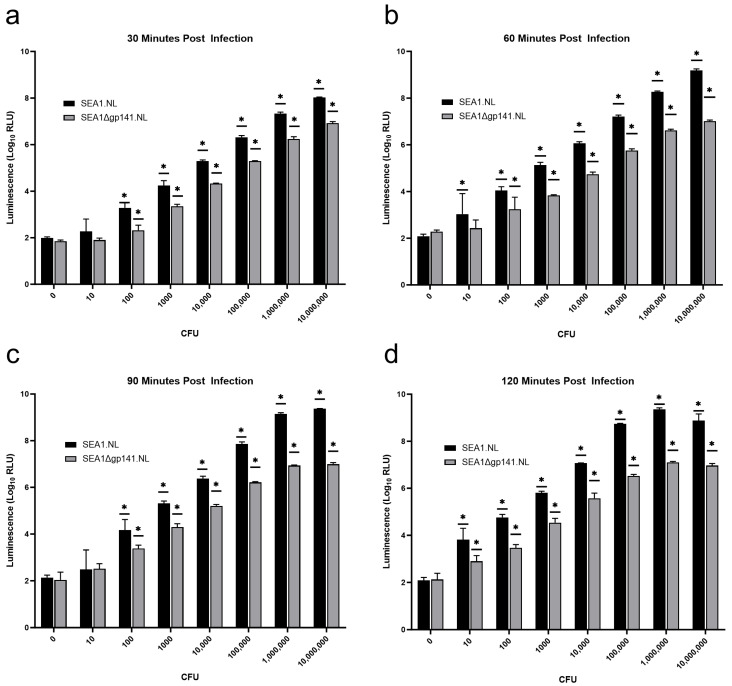
Both SEA1.NL and SEA1∆gp141.NL are capable of detecting *Salmonella*. Various colony-forming units (CFU) of *S. enterica* were infected for (**a**) 30 min, (**b**) 60 min, (**c**) 90 min, or (**d**) 120 min with either SEA1.NL or SEA1∆gp141.NL before being assessed for NanoLuc^®^ production. Luminescence was evaluated as log transformed mean relative light units (Log_10_ RLU) generated from triplicate wells. Error bars represent the standard deviation of these log-transformed values. Individual RLU values have been made available (Supplementary Table S2). Two-way ANOVAs with Dunnett’s multiple comparison tests were performed using GraphPad Prism. For each phage, asterisks denote a significant difference (*p* < 0.05) between the luminescence at that CFU and the corresponding background (0 CFU).

Due to all the indications of replication deficiency in the absence of gp141, it was of interest to determine the effectiveness of SEA1∆gp141.NL as a biocontained *Salmonella* diagnostic luciferase reporter phage. Thus, SEA1∆gp141.NL was used to infect various burdens of *S. enterica* for 30, 60, 90, or 120 min before being assessed for NanoLuc^®^ production. SEA1.NL was included to allow for side-by-side replication-competent comparison. SEA1.NL produced a robust signal over background from an inoculum of 100 colony-forming units (CFU) with only a 30-min infection (Figure 5a). Longer infections improved the signal and, with a two-hour infection, SEA1.NL yielded a significant signal from only 10 CFU (Figure 5b–d). These results agree with the previously reported sensitivity of SEA1.NL [12]. SEA1∆gp141.NL, like SEA1.NL, generated a significant signal over background from 100 CFU with a 30-min infection (Figure 5). Similarly, this improved with longer infection times, and a two-hour infection also produced a significant signal from 10 CFU. Despite a qualitative match, SEA1∆gp141.NL did yield a diminished signal across nearly every combination of burden and infection time when compared to SEA1.NL. Likely due to the absence of multiple rounds of infection, larger quantitative differences were generally found with higher bacterial burdens and longer infection times. Despite this modest reduction in signal, the overall performance of SEA1∆gp141.NL indicates that, even in the absence of phage replication, this reporter was capable of rapid, sensitive, and robust *Salmonella* detection.

## 4. Discussion

Although naturally occurring lytic phages are generally recognized as safe, the safety of genetically engineered phages is contingent on the underlying genetic modification and payload [41]. An extensive repertoire of foreign genes encoding diverse functions, such as nucleases, antimicrobial peptides, hydrolases, luciferases, and fluorescent proteins, have been engineered into phage genomes to achieve improved therapeutic efficacy and diagnostic utility [1,2,8,42,43]. In addition to active payloads, genetic engineering has also been used to improve upon and modify receptor-binding proteins, generating synthetic phages with unique and tailored host ranges [5,6]. The inadvertent release of genetically enhanced phages into the environment could have unintended side effects such as disrupting bacterial communities and promoting the development of resistance to either the phage or its payload [17,18]. Moreover, there is a risk of site contamination for any laboratory conducting phage-based diagnostics as even a few accidentally dispersed phages could multiply and interfere with bacterial isolation, culture enrichment, and downstream testing [44,45]. Further work to understand and address these concerns will be beneficial prior to a wide-spread use of synthetic phage-based technologies.

To achieve biocontainment, T7-like podoviruses have previously been made replication-deficient through the deletion of essential capsid or tail genes while supplying these proteins in trans for laboratory production [17]. To extend upon these findings, a biocontained *Salmonella* diagnostic reporter phage was pursued. Due to the extensive structural differences between T7-like podoviruses and SEA1, a T4-like myovirus, homologs to the tail genes previously targeted were not readily available [12,28,46]. Additionally, the major capsid protein of T4 is highly expressed to accommodate nearly 1000 copies per phage and may aggregate in the absence of additional phage proteins, such as the T4 chaperone [25,47]. These features were expected to reduce the efficiency of in trans complementation. As an alternative, a small essential baseplate wedge subunit (gp53), required at only six copies per phage, was a promising candidate [27]. The homolog of T4’s gp53 in SEA1 was found to be gp141 (Figure 1). Homologous recombination mediated the exchange of gp141 expression for NanoLuc^®^ expression in SEA1, creating SEA1∆gp141.NL, which was then isolated based upon luminescence, manufactured in an *S. enterica* strain expressing gp141 in trans and confirmed by PCR (Figure 2 and Figure 3a). Due to the number of promising T4-like lytic phages being investigated, the approach detailed in this study may serve as a useful blueprint to improve the safety profile across many phage-based applications [48,49,50].

In the absence of gp141, SEA1∆gp141.NL failed to form individual plaques at low titers, which is expected for a replication-deficient phage (Figure 3b). In contrast, persistent spot lysis was observed at higher phage concentrations. Although replication-deficient viruses lacking essential genes do not produce infectious progeny, they are still themselves infectious [51]. Replication-deficient phages are thus expected to normally adsorb, infect, and lyse host bacterial cells through the standard production of phage proteins, including holins, endolysins, and spannins [52]. In support of this, replication-deficient phages have been found to maintain antimicrobial activity and therapeutic efficacy in phage therapy [17]. Thus, at high titers, spot lysis may simply represent a lytic outcome of a single round of infection with the parent phage. Alternatively, this phenotype has previously been observed with both other replication-deficient phages and lysis-deficient phages incapable of producing endolysin [17,53]. These prior studies have suggested an alternative mechanism of spot lysis, “lysis from without”. In T4-like phages, lysis from without is a mechanism of phage-mediated bacterial lysis that does not require de novo phage protein production. It is restricted to high multiplicity of infection conditions and involves the extracellular activity of a tail lysozyme [54]. Since SEA1 does encode for a homolog of T4’s tail lysozyme, it is plausible that lysis from without could also contribute to spot lysis at high phage concentrations (Figure 1). Importantly, both mechanisms are not associated with productive infection and have little impact on the focus of this study, synthetic phage safety, and biocontainment.

A one-step growth curve was used to confirm that SEA1∆gp141.NL was replication-deficient and unable to produce infectious progeny without gp141 (Figure 4). These data also confirmed the efficacy of in trans expression of gp141 as SEA1∆gp141.NL demonstrate robust replication following infection of a complementing strain. Critically, no increase in titer was observed when SEA1∆gp141.NL infected a non-complementing *S. enterica* host. In fact, the total phage recovered at every time point after initial adsorption was well-below the starting inoculum despite PFU being evaluated on a *S. enterica* strain expressing gp141. Thus, as expected for replication-deficient viruses, the SEA1∆gp141.NL infection of native hosts in the absence of gp141 was a dead end, resulting in both the death of the parent phage and the absence of infectious progeny [51]. These data support the use of gp141 as an excellent target for the biocontainment of synthetic T4-like phages.

Investigations into the performance of SEA1∆gp141.NL as a *Salmonella* diagnostic reporter revealed decreased luminescence compared to SEA1.NL in nearly every condition (Figure 5). As expected, a larger difference was generally found in conditions with longer infection times and higher bacterial burdens where subsequent rounds of infection with infectious progeny are likely generating additional signal. However, this explanation is insufficient to explain the difference observed at low bacterial concentrations or the difference observed with a 30 min infection. A 30 min infection represents approximately the time required to complete only a single phage life cycle (Figure 4). Additional differences between SEA1.NL and SEA1∆gp141.NL, besides phage replication, may play a role here. For example, although both reporters contain the gene encoding NanoLuc^®^ under the same bacterial promoter, these sequences were inserted at two distinct genomic locations. The original reporter, SEA1.NL, inserted the promoter and reporter gene downstream of the gene encoding the major capsid protein [12]. In contrast, SEA1∆gp141.NL was created by exchanging the promoter and reporter gene with the sequence encoding a baseplate wedge subunit. Due to the distinct genomic context, additional elements, such as the upstream major capsid protein promoter, could be increasing NanoLuc^®^ production in SEA1.NL but not SEA1∆gp141.NL, thereby explaining these data. For this initial study, exchanging gp141 expression for NanoLuc^®^ expression was a straightforward approach to generating a replication-deficient reporter. Future iterations may benefit from removing gp141 expression while maintaining the original SEA1.NL location of the reporter gene.

Despite the reduced signal and absence of phage replication, SEA1∆gp141.NL remained an effective reporter for *Salmonella* contamination (Figure 5). Luminescence was burden-dependent and significantly different from the background at either 10 CFU with a 120 min infection or 100 CFU with a 30 min infection. Qualitatively, these results were similar to the performance of the replication-competent SEA1.NL found in this study and in prior work [12]. SEA1∆gp141.NL likely benefits from the high concentration of reporter phage utilized in this method. In this context, NanoLuc^®^ production from low burdens of *Salmonella* is likely already restricted to a single round of infection. Similarly, high burdens of *Salmonella* can be readily detected in 30 min, producing sufficient signal above background for detection from a single round of infection. Thus, this approach may be particularly well-suited to tolerate replication-deficient phages, regardless of bacterial burden.

In conclusion, these data indicate that homologs of the essential baseplate wedge subunit are suitable targets for the generation of bio-contained T4-like phages. Additionally, replication-deficient phages encoding NanoLuc^®^ are still capable of excellent performance as diagnostic reporters of bacterial contamination. Importantly, high titer stocks of replication-deficient phage reporters can be easily generated in the laboratory with complementing strains, maintaining the ease of production associated with many phage-based applications. Overall, this work affirms the suitability and functionality of bio-contained replication-deficient synthetic phages in phage-based diagnostics and may further be used as a blueprint to address safety concerns when genetically engineering other T4-like myoviruses.

## Data Availability

Data presented in this study are available within the article and supplementary material except for the genome sequence and annotation of SEA1. This information was recently submitted to GenBank^®^ (OQ927978) and is publicly available. Availability of the engineered bacteriophages described in this study may require a material transfer agreement covering potential commercial applications.

## Supplementary Materials

The following supporting information can be downloaded at: https://www.mdpi.com/article/10.3390/v16010008/s1, Figure S1: Original gel image for PCR confirmation of SEA1∆gp141.NL; Table S1: Plaque Counts for Figure 4; Table S2: Relative Light Units (RLU) Values for Figure 5.
