# Supplementary material for: Development of a Replication-Deficient Bacteriophage Reporter Lacking an Essential Baseplate Wedge Subunit
Source: Viruses. 2023 Dec 20;16(1):8. doi: 10.3390/v16010008 (PMC10821221; doi:10.3390/v16010008)
Supplement: Supplementary file 1 [file viruses-16-00008-s001.zip › viruses-2727516-supplementary.pdf]

Development of a Replication-Deficient Bacteriophage Reporter Lacking an Essential Baseplate Wedge Subunit

Jose Gil [1], John Paulson [2], Henriett Zahn [2], Matthew Brown [3], Minh M. Nguyen [2], and Stephen Erickson [2,*]

[1] Laboratory Corporation of America Holdings, Los Angeles, CA 90062, USA
[2] Laboratory Corporation of America Holdings, New Brighton, MN 55112, USA
[3] Laboratory Corporation of America Holdings, Burlington, NC 27215, USA
* Correspondence: erickss@labcorp.com

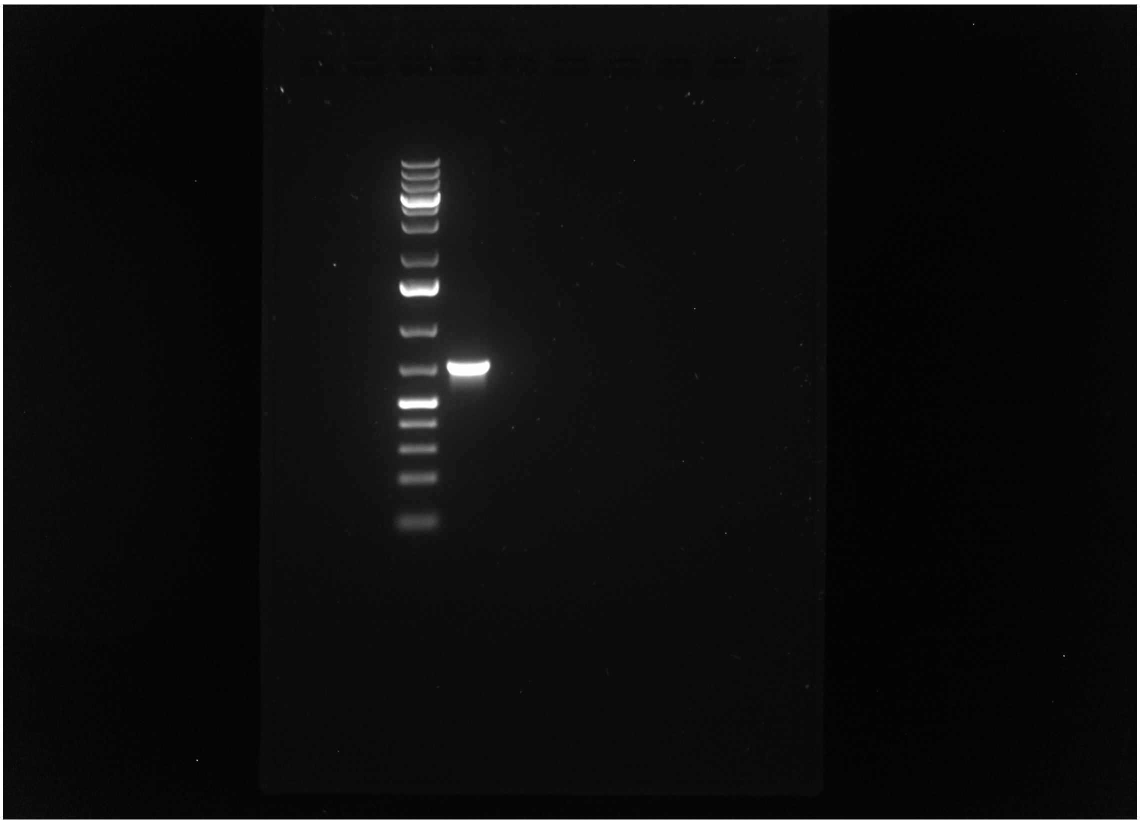

Figure S1. Original (uncropped) gel image for PCR confirmation of SEA1Δgp141.NL. PCR analysis was performed on DNA preparations from SEA1.NL and SEA1Δgp141.NL using primers specific for gp141. See Figure 3a in the main text for a cropped and annotated version. Image captured with a Gel Doc™ EZ Imager (Bio-Rad Laboratories, Hercules, CA, USA) running Image Lab Software (Bio-Rad Laboratories, Hercules, CA, USA).

Table S1. Plaque Counts for Figure 4

Phage	Host For Growth Curve	Host For Plaque Formation	Time (min)	Plaque Count[1]	Total PFU[2]
SEA1.NL	*S. enterica*	*S. enterica*	10	5	500
			15	8	800
			20	7	700
			25	6	600
			30	82	8200
			35	205	20,500
			40	414	41,400
			45	424	42,400
			50	588	58,800
			55	519	51,900
			60	508	50,800
			70	529	52,900
			80	588	58,800
			90	794	79,400
SEA1Δgp141.NL	*S. enterica*	*S. enterica*	10	0	ND
			15	0	ND
			20	0	ND
			25	0	ND
			30	0	ND
			35	0	ND
			40	0	ND
			45	0	ND
			50	0	ND
			55	0	ND
			60	0	ND
			70	0	ND
			80	0	ND
			90	0	ND
SEA1Δgp141.NL	*S. enterica*	*S. enterica* + pUC57.Comp.gp141	10	0	ND
			15	1	100
			20	0	ND
			25	0	ND
			30	1	100
			35	0	ND
			40	1	100
			45	0	ND
			50	0	ND
			55	0	ND
			60	0	ND
			70	0	ND
			80	0	ND
			90	0	ND
SEA1Δgp141.NL	*S. enterica* + pUC57.Comp.gp141	*S. enterica* + pUC57.Comp.gp141	10	3	300
			15	3	300
			20	3	300
			25	36	3600
			30	115	11,500
			35	154	15,400
			40	159	15,900
			45	178	17,800
			50	199	19,900
			55	185	18,500
			60	170	17,000
			70	162	16,200
			80	159	15,900
			90	185	18,500

[1] Samples were taken at the indicated time post infection of the indicated growth curve host with the indicated phage and plated for plaque forming units (PFU) on the indicated plaque formation host. [2] The limit of detection for this method was 100 total PFU. Values below this are marked not detected (ND).

Table S2. Relative Light Units (RLU) Values for Figure 5

Time (min)	Reporter[1]	CFU[2]	Well 1[3]	Well 2[3]	Well 3[3]
30	SEA1.NL	0	89	97	110
		10	95	93	769
		100	3402	1415	1483
		1000	18,996	10,405	26,835
		10,000	193,388	166,517	219,406
		100,000	2,391,743	1,728,102	2,162,510
		1,000,000	24,317,826	18,751,103	22,537,107
		10,000,000	108,984,104	109,798,332	103,876,296
	SEA1Δgp141.NL	0	79	63	73
		10	66	75	99
		100	292	268	116
		1000	2713	1793	2280
		10,000	20,713	21,443	22,189
		100,000	182,173	195,073	204,391
		1,000,000	1,311,912	2,011,278	1,947,802
		10,000,000	6,966,012	9,075,915	9,165,933
60	SEA1.NL	0	102	120	150
		10	4847	109	2407
		100	9142	16,956	9036
		1000	154,936	98,564	162,252
		10,000	1,372,072	1,126,496	1,024,891
		100,000	18,087,287	16,941,758	13,579,220
		1,000,000	204,001,256	178,745,640	181,321,320
		10,000,000	1,656,278,720	1,707,652,928	1,305,325,888
	SEA1Δgp141.NL	0	221	195	158
		10	515	350	109
		100	809	6828	958
		1000	6292	6832	7300
		10,000	43,799	61,365	63,014
		100,000	468,687	596,868	650,049
		1,000,000	3,595,047	4,621,079	4,181,567
		10,000,000	10,805,696	11,236,214	9,193,990
90	SEA1.NL	0	112	129	179
		10	103	97	2754
		100	4835	16,649	38,781
		1000	184,393	180,095	267,138
		10,000	2,124,725	2,096,262	3,084,814
		100,000	64,932,680	65,267,258	90,887,212
		1,000,000	1,296,987,136	1,353,762,112	1,593,387,264
		10,000,000	2,400,130,560	2,351,836,608	2,364,236,352
	SEA1Δgp141.NL	0	264	74	66
		10	524	343	198
		100	3321	1689	2327
		1000	22,216	25,395	13,476
		10,000	187,973	148,763	146,704
		100,000	1,691,484	1,518,846	1,701,398
		1,000,000	8,193,574	8,370,005	9,145,152
		10,000,000	10,674,117	10,761,234	8,127,210
120	SEA1.NL	0	100	107	170
		10	2474	21,786	5182
		100	42,101	61,660	74,499
		1000	595,452	564,964	765,981
		10,000	11,393,379	11,560,336	11,989,371
		100,000	518,650,672	580,298,752	535,321,088
		1,000,000	2,577,768,960	1,965,102,720	2,379,218,432
		10,000,000	358,969,104	1,194,215,456	1,004,905,408
	SEA1Δgp141.NL	0	254	78	119
		10	931	428	1237
		100	2761	2120	4093
		1000	21,043	39,514	47,658
		10,000	250,466	325,484	657,217
		100,000	3,066,944	3,030,819	3,961,988
		1,000,000	12,902,685	13,662,171	11,487,754
		10,000,000	8,260,648	8,308,753	11,613,242

[1] Phages were added to a concentration of 1 x 10^6 pfu/mL in each well. [2] Colony forming units (CFU) per well of *S. enterica*. [3] Relative light units (RLU) for triplicate wells are provided for each condition. Each well value is the average of two back-to-back measurements on a GloMax® Navigator.
